# From validation to public health impact: implementing equitable AI-enabled diabetic retinopathy screening

**DOI:** 10.3389/fpubh.2026.1976572

**Published:** 2026-10-05

**Authors:** Jing Chen, Bin Chen

**Affiliations:** Department of Ophthalmology, People's Hospital of Leshan, Leshan, Sichuan, China

**Keywords:** artificial intelligence, diabetic retinopathy, health equity, implementation science, public health, quality of life, referral completion, screening

## Abstract

Diabetic retinopathy (DR) remains a preventable cause of vision loss as diabetes prevalence rises worldwide. Artificial intelligence (AI)-enabled retinal screening has progressed from retrospective validation to prospective and real-world deployment, with recent reviews showing high diagnostic performance across fundus photography, smartphone-based systems, and autonomous platforms. Yet diagnostic accuracy alone does not ensure public health benefit. Population impact can be lost when eligible people are not reached, images are ungradable, results are delayed, referrals are incomplete, treatment capacity is insufficient, or disadvantaged groups face financial, geographic, or digital barriers. Unlike reviews centered mainly on diagnostic accuracy or economic performance, this Mini Review reframes AI-enabled DR screening as a population-health intervention spanning detection through completion of effective eye care. We synthesize evidence on diagnostic validity, implementation, economics, referral completion, care cascades, patient-centered outcomes, equity, and governance. We argue that effectiveness and cost-effectiveness are properties of the complete screening-to-treatment pathway, not the algorithm alone, and propose a four-domain framework encompassing technical validity, service integration, population impact, and equity and sustainability. Future research should prioritize comparative implementation studies and outcome sets that capture screening reach, gradability, referral completion, treatment initiation, time to treatment, avoidable vision loss, vision-related quality of life, acceptability, costs, and subgroup equity. The policy question is no longer whether AI can detect DR, but whether health systems can translate automated detection into equitable completion of effective eye care at scale.

## Introduction

Diabetic retinopathy (DR) is a common microvascular complication of diabetes and a preventable cause of vision loss when sight-threatening disease is detected and treated early (1). The public health challenge is expanding as diabetes prevalence rises: the International Diabetes Federation estimates that 589 million adults were living with diabetes in 2024, with projections approaching 853 million by 2050 (2). Updated global estimates suggest that more than 100 million adults already have DR, and the number is expected to increase substantially by 2045 (3). In this context, screening is not simply an ophthalmic service; it is a population-level strategy that links chronic disease management, primary care, imaging infrastructure, referral pathways, and timely treatment (4).

The 2016 review by Ting, Cheung, and Wong framed DR as a public health problem shaped by inadequate screening coverage, shortages of trained eye-care personnel, cost, uneven access, and limited service capacity in low-resource settings (1). Those barriers remain central. Teleophthalmology and digital health can help address distance and workforce constraints, but the World Health Organization has cautioned that digital interventions do not substitute for functioning health systems and require governance, training, infrastructure, and equity safeguards (5). During the past decade, AI-enabled DR screening has moved from landmark retrospective validation studies to prospective evaluations and real-world programmatic deployment (6–15). As this evidence has matured, the key question has shifted from whether algorithms can classify retinal images accurately to whether health systems can use automated detection to deliver completed, timely, and equitable eye care. This distinction matters because the public health value of AI is produced not at the image-classification step alone, but through the surrounding service pathway, including image acquisition, result communication, referral coordination, treatment capacity, patient engagement, and governance. Unlike reviews centered primarily on diagnostic accuracy or economic evaluation, this Mini Review examines AI-enabled DR screening through a public health implementation lens and evaluates the conditions required for algorithmic performance to translate into patient and population benefit.

## Literature search and selection criteria

This Mini Review was guided by the Scale for the Assessment of Narrative Review Articles (SANRA) framework, with particular attention to the justification of the review topic, clarity of aims, transparent description of literature selection, appropriate referencing, and scientific reasoning (16). A structured narrative literature search was conducted in PubMed, Web of Science, and Embase to identify peer-reviewed articles and relevant policy or regulatory documents addressing AI-enabled diabetic retinopathy screening. The search focused on literature published between January 2016 and August 2026, reflecting the period from landmark deep learning validation studies to recent real-world implementation, health economic, equity, and governance evidence. Foundational sources published in 2016 were retained when they informed diabetic retinopathy screening policy, public health framing, or digital health implementation principles.

Search terms included combinations of “diabetic retinopathy,” “artificial intelligence,” “deep learning,” “screening,” “teleophthalmology,” “autonomous AI,” “fundus photography,” “diagnostic accuracy,” “real-world validation,” “implementation,” “referral completion,” “care cascade,” “cost-effectiveness,” “quality of life,” “health equity,” “bias,” “regulation,” and “governance.” Priority was given to systematic reviews, meta-analyses, prospective validation studies, real-world implementation studies, randomized or pragmatic evaluations, health economic analyses, patient-centered outcome studies, and policy or regulatory documents directly relevant to the transition from algorithm validation to public health implementation. Reference lists of key reviews and implementation studies were also screened to identify additional relevant sources. Because this was a Mini Review rather than a systematic review, formal risk-of-bias assessment, exhaustive study inclusion, and quantitative evidence synthesis were not performed.

## What has diagnostic validation established?

The diagnostic evidence base for AI-enabled DR screening is now substantial. Early work by Gulshan and colleagues showed that deep learning could detect referable DR from retinal fundus photographs with high sensitivity and specificity in curated validation datasets (6). Ting and colleagues extended this work to multiethnic retinal image datasets and demonstrated performance across DR and other ocular conditions (7). Abramoff and colleagues then reported a pivotal prospective trial of an autonomous diagnostic system in primary care offices, supporting the clinical feasibility of point-of-care AI screening without specialist interpretation (8). These studies established proof of concept: DR is a suitable target for automated image interpretation because screening uses structured retinal images, standard disease thresholds, and clear referral decisions. More recent reviews clarify what has and has not been established in practice. Joseph and colleagues synthesized 34 real-world studies and reported pooled sensitivity of 94% and specificity of 89% among gradable images, with income level and fundus camera type explaining part of the heterogeneity (12). Ran and colleagues reviewed 47 prospective validation studies and found high discriminative performance, particularly for vision-threatening DR, while also showing that region, clinical pathway, mydriasis, image quality control, sample size, grading criteria, reference standard, and model architecture affected performance (15). Taken together, these studies indicate that AI diagnostic performance for DR screening is broadly credible, particularly when image acquisition, quality assurance, and referral thresholds are well specified.

However, diagnostic validation is not interchangeable with program effectiveness. Smartphone-based AI may expand access in low-resource or mobile settings, and Hasan and Siddiqui reported high diagnostic accuracy for smartphone-based systems, especially for referable DR (13). Product-specific evidence for IDx-DR also suggests strong pooled accuracy, but performance is tied to the device, protocol, target disease definition, and intended-use population (14). Independent head-to-head validation has shown that performance can vary materially across algorithms even when systems are applied to the same real-world data (10). Real-time national deployment in Thailand and African clinical validation studies further show that AI performance depends on workflow, image acquisition, and the surrounding screening pathway (9, 11). Diagnostic validation should therefore be viewed as a prerequisite for, rather than a substitute for, evaluation of program effectiveness. Real-world performance remains contingent on equipment, image quality, population, and pathway design; analyses restricted to gradable images may overestimate program performance; and public health benefit depends on linking detection to completed eye care. The mature validation literature now provides a basis for implementation studies that specify the model, target population, camera, disease threshold, and pathways for non-diagnostic, positive, and urgent results.

## The implementation gap: accuracy is not population effectiveness

A high-performing algorithm can lose much of its public health value as patients move through the screening-to-treatment pathway. The central issue is a shift in denominator: diagnostic studies often evaluate gradable images, whereas screening programs are accountable for the eligible population with diabetes. Program effectiveness may therefore be diluted before, during, and after image interpretation. Before imaging, limited invitation, low attendance, or poor access can reduce screening reach. During imaging, ungradable or non-diagnostic images remain part of the service burden even when they are excluded from accuracy estimates. After AI interpretation, delayed result communication, unclear referral pathways, insufficient patient-facing communication, referral loss, or inadequate treatment capacity can prevent automated detection from becoming completed care (17–23). In this sense, AI performance is not lost at a single point; it is progressively translated, weakened, or preserved across the care cascade.

This pathway perspective is supported by implementation and care-cascade evidence. In Thailand, a digital platform integrating AI had to transform non-digital workflows, establish tracking, and coordinate follow-up for vision-threatening DR (17). The RAIDERS randomized trial in Rwanda showed that AI assessment could improve referral service uptake in a low-resource setting when results and referral processes were embedded in an intervention rather than treated as a stand-alone test (18). Bresnick and colleagues showed that even after primary care DR screening, adherence to ophthalmology referral, treatment, and follow-up can remain incomplete (19). Dow and colleagues reported improved follow-up in an AI-based workflow, but the result was linked to workflow differences, result timing, and patient communication rather than the algorithm alone (20). The ACCESS randomized trial showed that autonomous AI increased diabetic eye exam completion among youth, but also highlighted the need to measure follow-through after abnormal results (21). A 2026 meta-analysis of AI-assisted pathways found improved referral uptake, but with high heterogeneity and low certainty, emphasizing that pathway redesign and patient-facing interventions drive much of the observed effect (22). For public health planning, the relevant unit of analysis is therefore the complete care cascade. A screening program should report how many eligible patients are reached, how many images are ungradable, how many positive results lead to completed referral, how quickly treatment begins, and whether outcomes improve. These measures should be reported as linked proportions rather than isolated process indicators, because a strong result at one stage can be neutralized by failure at the next. Without these data, AI may increase detection without reducing avoidable vision loss.

## Health-system and economic conditions for implementation

Economic evidence for AI-enabled DR screening should be interpreted as pathway evidence, not algorithm evidence. Ran and colleagues found that deep learning-based DR screening appeared generally favorable in high-income countries, whereas results in middle-income countries were mixed and sensitive to compliance rates, baseline glycemic control, and initial implementation costs; evidence from low-income countries remains sparse (15). This pattern is consistent with the broader digital health principle that effectiveness depends on infrastructure, governance, workforce, and integration into existing services (5).

Context-specific economic studies reinforce this point. In urban China, Lin and colleagues found that an AI-assisted community telemedicine model was not automatically cost-effective compared with manual grading, but its value improved when referral compliance increased (24). Wang and colleagues showed that the most diagnostically accurate model is not necessarily the most cost-effective option; the preferred trade-off between sensitivity and specificity depends on prevalence, willingness-to-pay thresholds, costs of false positives, costs of missed disease, and downstream treatment capacity (25). Recent health economic reviews similarly argue that evaluations should incorporate local adherence, regional cost data, and transparent reporting of pathway assumptions (26).

Autonomous point-of-care, centralized AI or teleophthalmology, and hybrid AI-plus-human grading models should not be ranked universally, because each offers a different balance of immediacy, specialist oversight, infrastructure requirements, and workflow complexity. Autonomous systems can provide same-visit results and reduce dependence on asynchronous grading, whereas centralized models may support multisite quality assurance and specialist oversight; hybrid models retain human review for selected or uncertain cases (8, 10, 11, 14, 17, 21). Their suitability therefore depends on local workforce, connectivity, image-quality management, referral and treatment capacity, governance, and financing, with the principal implementation requirements and risks summarized in Table 1. Operational protocols should also specify whether AI-negative patients return to routine screening, whether mild DR triggers intensification of diabetes management, and whether ungradable or non-diagnostic images are repeated, reviewed, or referred. Budget planning should likewise account for downstream referral and treatment capacity rather than screening throughput alone.

**Table 1:** Context-specific comparison of AI-enabled DR screening service models.

Service model	Potential service context	Key implementation requirements and risks
Autonomous AI point-of-care screening	Primary care, diabetes clinics, pharmacies, or mobile services where same-visit results may reduce delays in screening completion and follow-up.	Device-specific training and quality control, management of ungradable or non-diagnostic images, referral and treatment capacity, and clear accountability for abnormal results.
Centralized AI or teleophthalmology	Programs collecting images across multiple sites with centralized reporting, quality assurance, or specialist oversight.	Reliable image transfer and reporting infrastructure, turnaround time, management of ungradable or borderline cases, human workload for exceptions, and potential loss to follow-up before referral communication.
Hybrid AI-plus-human grading	Established screening programs using AI for triage or first-pass grading while retaining human review for selected cases.	Human-review workload, workflow and handoff complexity, reduced speed advantages, management of borderline cases, and clear allocation of responsibility between automated and human review.

Service contexts are illustrative rather than prescriptive and should be adapted to local workforce, infrastructure, referral and treatment capacity, and financing.

## From detection to patient-centered outcomes

Beyond diagnostic and economic endpoints, patient-centered outcomes are central to evaluating the public health value of AI-enabled DR screening. Screening programs often evaluate sensitivity, specificity, throughput, and cost per case detected. These are important intermediate outcomes, but they do not capture whether patients experience better vision, less functional limitation, lower anxiety, or more acceptable care. Zayed and colleagues' systematic review and meta-analysis found that DR severity is associated with a graded decline in vision-related quality of life (VRQoL), and that even non-vision-threatening DR may be associated with worse VRQoL (27). The review also suggested that generic health-related quality-of-life instruments may be less sensitive than vision-specific measures, and that visual acuity alone does not fully explain patient experience. These findings matter for AI screening because the purpose of earlier detection is not merely to identify retinal lesions, but to prevent disability and protect daily functioning.

A public health evaluation of AI-enabled DR screening should therefore include referral completion, treatment initiation, time to treatment, avoidable vision loss, VRQoL, screening-related anxiety, and patient acceptability. These outcomes are particularly important when AI provides same-visit results, shifting screening from passive image acquisition to immediate risk communication. Same-day feedback may support follow-up only when patients understand what a positive, negative, or non-diagnostic result means and receive clear counseling, referral explanation, and navigation. Without these supports, rapid notification may increase uncertainty or anxiety rather than improve the screening experience. Patient acceptability should therefore be evaluated alongside referral adherence and satisfaction, rather than treated as an incidental feasibility finding (28, 29). The downstream evidence is still thinner than the diagnostic evidence. Referral uptake has improved in some AI-assisted pathways, yet few studies follow patients through treatment and visual outcomes (19–23). Recent care-cascade evidence shows persistent losses across screening, diagnosis, care adherence, and blindness prevention (23). Future studies should therefore evaluate whether AI shortens the time from screening to treatment and whether this produces measurable gains in vision and VRQoL.

## Equity, regulation, and accountability

Equity should be treated as a primary implementation outcome, not a secondary ethical concern. AI-enabled screening could reduce disparities by moving retinal assessment into primary care, diabetes clinics, mobile units, pharmacies, or community settings. It may be particularly valuable for rural and remote populations, low-income patients, older adults, and people who face long travel distances or limited specialist availability (28–30). However, the same technology can reproduce or worsen inequity if devices are unavailable, internet connectivity is weak, staff are inadequately trained, costs are shifted to patients, or algorithms perform differently across populations and camera systems (10, 31–33).

Ungradable images are a central equity issue. Cataract, small pupils, media opacity, older age, and poor imaging conditions may be more common in groups already at higher risk of limited access. Excluding ungradable images from diagnostic accuracy analyses can make a program look safer and more effective than it is. Ungradable images should therefore be treated as an implementation and equity outcome, not merely a technical exclusion. Programs should report ungradable rates by site, device, age, race or ethnicity where appropriate, ocular comorbidity, and deprivation or rurality indicators, and should specify whether ungradable results trigger repeat imaging, dilation, human review, or direct referral.

Regulatory and governance requirements should remain proportionate and globally adaptable. Multicenter external validation, fixed algorithm-version documentation, revalidation after updates, post-market performance monitoring, data-drift surveillance, and evaluation of device combinations are necessary to maintain safety over time (10, 32–35). FDA-cleared autonomous systems provide useful examples, but the US approval pathway should not be treated as a universal template. Countries and regions need regulatory approaches that match local infrastructure, resource constraints, accountability arrangements, and public health priorities. Responsibility should be explicit across developers, device manufacturers, clinical sites, screening programs, payers, and health authorities, especially when software updates, local workflow changes, or new camera combinations alter real-world performance.

## A public health implementation framework

We propose a four-domain framework for evaluating AI-enabled DR screening as a public health intervention (Figure 1). The domains are interdependent rather than sequential, because weakness in any one domain can limit population benefit. Technical validity includes diagnostic accuracy, gradability, external validation, calibration, image-quality assessment, device compatibility, and performance across clinically relevant subgroups. It asks whether the AI system works for the intended population, disease threshold, camera, imaging protocol, and clinical workflow (10, 12, 15, 32, 33). Service integration includes workflow, staffing, training, result communication, human review, referral criteria, data transfer, electronic health record integration, and treatment capacity. It asks whether screening can be delivered reliably and whether abnormal or non-diagnostic results lead to a clear next step (17, 20, 22).

**Figure 1:**
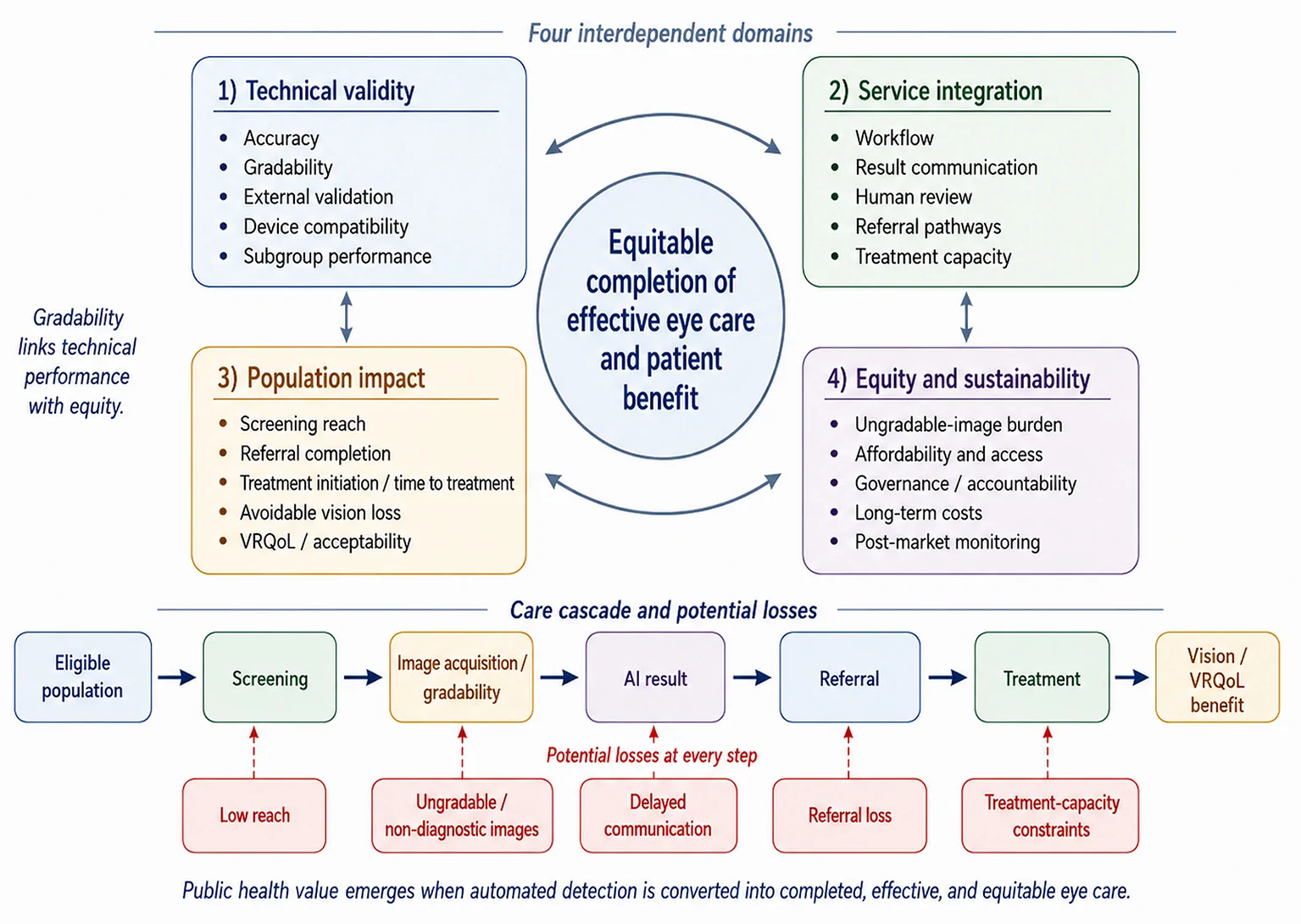
From diagnostic validation to equitable completion of care: a public health implementation framework for AI-enabled diabetic retinopathy screening. The framework illustrates how diagnostic performance must be translated across four interdependent implementation domains: technical validity, service integration, population impact, and equity and sustainability. Together, these domains support equitable completion of effective eye care and meaningful patient benefit. The lower care cascade traces the pathway from the eligible population through screening, image acquisition and gradability assessment, AI-generated results, referral, and treatment to vision- and quality-of-life benefit. Red boxes indicate potential losses at successive stages, including limited screening reach, ungradable or non-diagnostic images, delayed result communication, referral loss, and treatment-capacity constraints. Gradability is positioned as a cross-cutting implementation issue because image acquisition failure can affect both technical performance and equitable access to downstream care. The framework emphasizes that the public health value of AI-enabled screening depends not only on diagnostic accuracy but also on successful completion of the full care pathway.

Population impact includes screening coverage, attendance, referral completion, treatment initiation, time to treatment, avoidable vision loss, and VRQoL. It asks whether the program improves outcomes that matter to patients and health systems, not only whether it classifies images accurately (22, 23, 27). Equity and sustainability include subgroup performance, ungradable images, affordability, rural and remote access, long-term costs, governance, data protection, accountability, and post-market monitoring (26, 29, 31, 34, 35). It asks whether benefits are distributed fairly and whether the program can be maintained over time. This framework shifts evaluation from the narrow question of whether the algorithm is accurate to the broader public health question of under what conditions, for whom, and through which pathway AI-enabled DR screening produces equitable completion of effective eye care.

## Discussion

Current evidence supports context-specific implementation of AI-enabled DR screening, but not indiscriminate scale-up. The main implication of this Mini Review is that AI screening should be evaluated as a service intervention rather than as an isolated diagnostic test. Diagnostic validation remains essential, but implementation decisions should also account for local image quality, camera systems, workflow design, referral pathways, treatment capacity, patient-facing communication, equity safeguards, and ongoing performance monitoring (15, 22, 23, 31, 34). This distinction is clinically and programmatically important: an AI system may increase case detection, but preventable vision loss will not decrease if abnormal or non-diagnostic results do not lead to timely specialist assessment, treatment initiation, visual benefit, and acceptable patient experience.

Future research should therefore move beyond retrospective accuracy studies toward comparative implementation studies. Pragmatic trials, interrupted time-series analyses, prospective cohorts, and embedded health economic evaluations should compare service models, not only algorithms. Studies should prespecify the denominator at each stage of the pathway, include ungradable and non-diagnostic images in program-level analyses, and follow positive or non-diagnostic results through the next clinically meaningful action (22, 23). For public health evaluation, outcome reporting should span the full care pathway, including reach, gradability, referral completion, treatment initiation, time to treatment, vision-related quality of life, acceptability, costs, and subgroup equity. Reporting these outcomes together would provide a more complete assessment of program performance than isolated process indicators.

Implementation should also be locally adaptive. Performance and cost-effectiveness estimates from high-income settings should not be extrapolated uncritically to lower-resource settings, where imaging quality, labor costs, connectivity, referral barriers, treatment capacity, and financing may alter conclusions (15, 26). Similarly, equity and governance cannot be added after deployment; they must be built into model validation, site selection, workflow design, accountability arrangements, post-market monitoring, and plans for software or device changes (31, 34, 35). The next phase of AI-enabled DR screening should therefore prioritize implementation evidence that links automated detection to completed referral, treatment access, visual outcomes, patient experience, and equitable service delivery.
